# Safety Profile of Levonorgestrel: A Disproportionality Analysis of Food and Drug Administration Adverse Event Reporting System (Faers) Database

**Published:** 2018

**Authors:** Anitha Kurian, Kanika Kaushik, Viswam Subeesh, Eswaran Maheswari, Radhika Kunnavil

**Affiliations:** 1- Department of Pharmacy Practice, Faculty of Pharmacy, M.S. Ramaiah University of Applied Sciences, Bangalore, India; 2- Department of Community Medicine, M.S. Ramaiah Medical College, Bangalore, India

**Keywords:** Contraceptive, Data mining, Levonorgestrel

## Abstract

**Background:**

Levonorgestrel is most commonly utilized as an emergency oral contraceptive. Little is known and/or studied about the adverse effects of levonorgestrel, therefore, current investigation was aimed to generate signal for unreported adverse drug reactions of levonorgestrel using disproportionality analysis in food and drug administration adverse events reporting system database.

**Methods:**

In FDA Adverse Events Reporting System (FAERS) database, all adverse event reports for levonorgestrel between January 2006 to June 2015 were identified and disproportionality analysis was conducted for selected adverse events of levonorgestrel using Reporting Odds Ratio, Proportional Reporting Ratio and Information Component with 95% confidence interval.

**Results:**

A disproportionality analysis was done for 15 adverse events of levonorgestrel; out of these, signal for 10 adverse events was found and among them menstruation delayed was reported maximum (1791), followed by pregnancy after post-coital contraception (942), breast tenderness (901), metrorrhagia (899), dysmenorrhea (822), menorrhagia (541), nipple disorder (141), breast enlargement (77), ectopic pregnancy (61) and premenstrual syndrome (35). Pregnancy after post-coital contraception showed the highest signal having the Information Component value of 129.2, Reporting Odds Ratio value of 6.51 and Proportional Reporting Ratio value of 6.49.

**Conclusion:**

In this paper, ten novel AEs were identified that were disproportionately reported with the use of LNG by using data mining techniques. Although a causal relationship cannot be established, the number of cases reported suggests that there might be an association. If confirmed by epidemiologic studies, the findings from this study would have potential implications for the use of LNG and patient management in clinical practice.

## Introduction

Contraception or birth control prevents pregnancy by interfering with the normal process of ovulation, fertilization, and implantation (1–3). The methods of emergency contraception include Emergency Contraception Pills (ECPs), combined oral contraceptive pills or the Yuzpe method, Copper-bearing Intrauterine Devices (IUDs) (4, 5). Levonorgestrel (LNG) is a synthetic and biologically active progestogen that inhibits ovulation or fertilization and is used as an oral emergency contraceptive (EC) (6).

It is difficult to determine all the adverse effects of a drug in pre-licensure clinical trials due to restricted sample size, limited exposure time, reduced follow-up, exclusion of testing in special population and subjects with multiple co-morbidities. The need for post-marketing surveillance (PMS) is a direct result of these limitations. PMS is the identification and collection of safety information regarding medications after their approval by the U.S. Food and Drug Administration (FDA) which evaluates the drugs taken by individuals under a wide range of circumstances over an extended period of time (7, 8). It plays a major role in identifying previously unrecognized newer adverse events (AEs). Other important components of PMS include unapproved/off-label drug use, problems with orphan drugs and lack of paediatric formulations. Signal detection is one of the major aims of Pharmacovigilance (PV), *i.e*., the identification of potential drug-event associations that may be novel by virtue of their nature, severity and/or frequency. The WHO has defined “signal” as “Reported information on a possible causal relationship between an adverse event and a drug, the relationship being unknown or incompletely documented” (9, 10). Signal detection is used to identify previously unknown AEs of marketed drugs.

An emergency contraceptive like LNG is a reliable, simple and effective way to avoid unwanted pregnancy. The healthcare professionals and consumers are quite ignorant about the complications associated with the emergency contraception. Lack of awareness about the appropriate usage of LNG and easy availability as over the counter (OTC) drug, made it more vulnerable for serious AEs (11). Hence, our study aimed to generate signal for unreported adverse drug reactions of LNG using disproportionality analysis in food and drug administration adverse events reporting system (FAERS) database.

## Methods

### Data source:

AE reports from the FAERS database were used for the study. It is a surveillance program useful for detecting serious AEs not identified during premarketing analysis. Our study was based on all reports of levonorgestrel received by the FDA from 2006Q1-2015Q2(12).

### Study variables:

The information contained within FAERS report includes the name of one or more suspect drugs (Those suspected by the reporter of causing, or being causally related to, an AE), concomitant drugs, and patient characteristics (age and sex). An unlimited number of AEs can be reported in a single report (12). Each event was coded using the standardized terminology of the MedDRA^®^.

### Study procedure:

The FAERS database from 2006 Q1 to 2015Q2, each year consisting of 4 quadrants, Q1, Q2, Q3, and Q4 was utilized. Every quadrant file had details about the demographics, reactions, statistics, and drugs associated with the AE reported in that quadrant.

In the first step, case IDs of the LNG drug were isolated from drug file, out of which only primary and secondary reports were included, and others were excluded. These case IDs were transferred to the reaction file, which consisted of all the reactions of that quadrant. In the reaction file, using case IDs of LNG the corresponding reactions were sorted. The obtained reactions were arranged alphabetically and frequently reported AEs were listed and their frequency in each quadrant was noted.

Few repeatedly reported clinically relevant and rare ADRs were chosen and evaluated for IC, ROR and PRR. For the evaluation of each AE number of Ni (The event of interest), Nj (Drug of interest), Nij (Both events of interest and drug of interest) were listed. The further calculation was carried out for IC, ROR and PRR to find the correlation between LNG and AE.

### Statistical analysis:

The threshold for statistical significance was predefined as a PRR of ≥2.0 with a Chi-squared test statistic of ≥4.0, at least three reports (n ≥3) of that preferred term (PT), IC with IC-2SD>0 and ROR with ROR-1.96SE>1. Any PT that met all of these three criteria was disproportionally reported for levonorgestrel at a higher rate than expected (13). In order to evaluate PRR and ROR with 95% confidence interval (95% CI), the calculation was done using the equation ROR, CI=e^ln(ROR)±1.96^ and PRR, CI=e^ln(PRR)±1.96^, respectively. Positive signals were highlighted using bold letters. Detailed calculation of ROR, PRR and IC were discussed by Poluzzi et al. (9).

## Results

**Figure 1.**
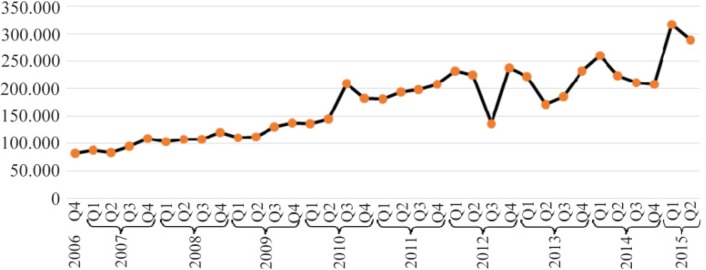
Number of reports in FDA AERS database

A total of 6,248,355 ADR reports were recorded during the period of 2006Q1 to 2015Q2 (38 Quarters) (Figure 1). Out of these, 43582 ADR reports were primarily or secondarily suspected by LNG.

### Data mining and clinical review:

A total of 837 PTs were identified by using data mining algorithms. Clinically relevant 15 PTs were selected for which signal detection process was performed.

The majority of PTs were reproductive system related disorders (Breast enlargement, breast tenderness, dysmenorrhea, ectopic pregnancy, menorrhagia, menstruation delayed, metrorrhagia, nipple disorder, pregnancy after post coital contraception, premenstrual syndrome) and the remaining were categorized under miscellaneous category (Diarrhoea, dysuria, fungal infection, muscle spasm, pollakiuria).

### Positive signals identified using ROR:

The ROR for 10 PTs ranged from 6.51 (95% CI, 6.34–6.67), being the highest for pregnancy post-coital contraception to 1.3 (95% CI, 1.04–1.55), being the lowest for ectopic pregnancy. The ROR values were as follows for the PTs menstruation delayed 4.5 (95% CI, 4.46–4.59), breast tenderness 3.7 (95% CI, 3.62–3.77), dysmenorrhea 3.46 (95% CI, 3.38–3.53), nipple disorder 2.83 (95% CI, 2.65–3), metrorrhagia 2.63 (95% CI, 2.56–2.69), menorrhagia 1.89 (95% CI, 1.8–1.97), breast enlargement 1.7 (95% CI, 1.47–1.92), and premenstrual syndrome 1.5 (95% CI, 1.16–1.83) (Table 1).

**Table 1.** Data mining algorithm values of all clinically relevant reactions associated with levonorgestrel use

**Reaction**	**IC**	**ROR**	**PRR**
**Breast enlargement**	1.16	1.7	1.7
**Breast tenderness**	12.38	3.7	3.68
**Dysmenorrhea**	9.72	3.46	3.44
**Ectopic pregnancy**	0.5	1.3	1.3
**Menorrhagia**	1.86	1.89	1.88
**Menstruation delayed**	28.24	4.53	4.49
**Metrorrhagia**	4.2	2.63	2.61
**Nipple disorder**	4.8	2.83	2.83
**Pregnancy after post coital contraception**	129.22	6.51	6.49
**Premenstrual syndrome**	0.52	1.5	1.5
**Diarrhoea**	−0.26	−1.36	−1.34
**Dysuria**	−0.37	−0.27	−0.27
**Fungal infection**	−0.25	0.08	0.08
**Muscle spasm**	−0.05	−0.18	−0.18
**Pollakiuria**	−0.13	0.16	0.16

ROR: Reporting Odds Ratio, PRR: Proportional Reporting Ratio, IC: Information Component

### Positive signals identified using PRR:

The PRR for 10 PTs ranged from 6.49 (95% CI, 6.32–6.65), being the highest for pregnancy post-coital contraception to 1.3 (95% CI, 1.04–1.55), being the lowest for ectopic pregnancy. The PRR values were as follows for the PTs menstruation delayed 4.49 (95% CI, 4.43–4.54), breast tenderness 3.68 (95% CI, 3.6–3.75), dysmenorrhea 3.44 (95% CI, 3.36–3.51), nipple disorder 2.83 (95% CI, 2.65–3), metrorrhagia 2.61 (95% CI, 2.54–2.67), menorrhagia 1.87 (95% CI, 1.79–1.96), breast enlargement 1.7 (95% CI, 1.47–1.92), and premenstrual syndrome 1.5 (95% CI, 1.16–1.83) (Table 1).

### Positive signals identified using IC:

The IC for 10 PTs ranged from 129.2 (95% CI, 128.67–129.76), being the highest for pregnancy post-coital contraception to 0.5 (95% CI, −0.35–1.35), being the lowest for ectopic pregnancy. The IC values were as follows for the PTs menstruation delayed 28.2 (95% CI, 28.03–28.44), breast tenderness 12.38 (95% CI, 12.12–12.63), dysmenorrhea 9.7 (95% CI, 9.46–9.97), nipple disorder 4.8 (95% CI, 4.20–5.39), metrorrhagia 4.2 (95% CI, 3.96–4.43), menorrhagia 1.87 (95% CI, 1.57–2.16), premenstrual syndrome 1.52 (95% CI, −0.61–1.65), and breast enlargement 1.16 (95% CI, 0.38–1.93) (Table 1).

## Discussion

The regulatory agencies and pharmaceutical industry use data mining to propose drug-AE pairs for further clinical review by screening large databases like FAERS. Data mining is used to identify previously unknown AEs of marketed drugs (12). In this study, ten novel AEs were identified that were disproportionately reported and potentially associated with the use of LNG, by using data mining techniques.

AE reported disproportionately for a particular drug can be identified by the application of data mining algorithms to spontaneous reports of AEs. Conversely, they do not establish a causal relationship between the drug and the event. Previous knowledge and potential mechanisms should be integrated during the clinical review to determine if the signal identified by data mining requires additional evaluation (12).

By applying data mining algorithms in spontaneous reports, a number of clinical case reports (Breast enlargement, breast pain, dysmenorrhea, ectopic pregnancy, menorrhagia, metrorrhagia, menstruation delayed, nipple disorder, pregnancy after post coital contraception, premenstrual syndrome) were found suggesting some justification for further analysis of a possible causal link with LNG.

AEs related to breast tissue such as breast enlargement, tenderness and nipple disorder showed a positive signal. Breast enlargement and breast tenderness could be due to edema and fluid retention which is caused by estrogen and progesterone in the breast tissues. Also, progesterone plays a role in lobular–alveolar structure formation. At the end of the luteal phase, there is an increase in mitosis of epithelial cells and hence DNA synthesis could cause an increase in breast cells. During clinical trials of Plan B one Step (LNG), 8.2% of total AEs were comprised of breast tenderness (14–16). Nipple discharge and nipple pain may be caused by progesterone effects in hypothalamus-pituitary-ovarian axis and its interference with prolactin inhibition by the hypothalamus. The tyrosine releasing hormone can enhance release of prolactin which can contribute to galactorrhea, the milky discharge from the nipple (17, 18).

In our study, increased reports of menstruation-associated AEs were found. Delayed menstruation (>7 days) [4.5% of AEs] and heavier menstruation (menorrhagia) [30% of AEs] were reported in Plan B One-Step clinical trials. Progestin-only pill cause variations in hypothalamus-pituitary-ovarian axis leading to changes in the secretion of gona-dotropin-releasing hormone (GnRH) from the hypothalamus. This produces alterations in the release of luteinizing hormone (LH) and follicle-stimulating hormone (FSH) delaying ovulation and release of progesterone from corpus luteum hence causing a delay in menses (19). Progesterone-estrogen balance is essential for the normal menstrual flow and any imbalance in the hormones results in increased endometrium development culminating in heavy menstrual bleeding (Menorrhagia) (20). Disproportionate reporting for a premenstrual syndrome was found which can also be attributed to an imbalance in estrogen and progesterone (21). Dysmenorrhea was identified as an AE during post-marketing surveillance. Progesterone levels fall in the late luteal phase, increasing the levels of prostaglandins (F2α) in the endometrium which stimulates myometrium and causes uterine contractions. Ischemia in the endometrium generates anaerobic metabolites, which increases the pain sensitivity in the uterus causing type C pain. This leads to the effects of dysmenorrhea (22, 23).

Ectopic pregnancy and pregnancy after post-coital contraception were found to have a co-relation with LNG, latter having a strong correlation. Alteration in the tubal motility causes a delay in the egg to reach the endometrium, leading to ectopic pregnancy. Failure of the progestin-only pill to inhibit ovulation by negative feedback mechanism on the hypothalamus and decrease in LH and FSH production can lead to pregnancy (24, 25).

Other AEs like diarrhoea, dysuria, fungal infection, muscle spasm, pollakiuria reported to FAERS had no positive signal till 2015Q2. This study was subject to certain limitations. It had been reported that when a drug first obtains marketing authorisation, there is a significant increase in spontaneous reporting of ADRs (Especially in the first two years), which then plateaus and finally declines; this phenomenon is known as Weber effect. Also, notoriety bias is defined as “A case has a greater chance of being reported if the subject is exposed to a factor known, thought or likely to cause the event of interest” or in other words, increased reporting for a drug following safety alert/labelling changes.

## Conclusion

Post-marketing studies help in determining potential safety issues. In this paper, ten novel AEs (Breast enlargement, breast tenderness, dysmenorrhea, ectopic pregnancy, menorrhagia, menstruation delayed, metrorrhagia, nipple disorder, pregnancy after post-coital contraception and premenstrual syndrome) were identified that were disproportionately reported and associated with the use of LNG by using data mining techniques. Although a causal relationship cannot be established, the number of cases reported suggests that there might be an association. If confirmed by epidemiologic studies, the findings from this study would have potential implications for the use of LNG and patient management in clinical practice. Healthcare providers should be vigilant about the possibility of encountering serious AEs identified in this analysis and should report them to the regulatory authorities. By implementing guidelines for dispensing oral contraception, and making it prescription only drug, the number of AEs with the same can be reduced in future.
